# *In vivo *solid tumor targeting with recombinant VEGF-diphtheria immunotoxin 

**DOI:** 10.22038/IJBMS.2021.54293.12195

**Published:** 2022-01

**Authors:** Mohammad Hosseininejad-Chafi, Ehsan Alirahimi, Behzad Ramezani, Akbar Oghalaie, Nazli Sotoudeh, Hajarsadat Ghaderi, Fatemeh Kazemi-Lomedasht, Mahdi Habibi-Anbouhi, Reza Moazzami, Mahdi Behdani

**Affiliations:** 1 Biotechnology Research Center, Venom & Biotherapeutics Molecules Lab, Pasteur Institute of Iran, Tehran, Iran; 2 National Cell Bank, Pasteur Institute of Iran, Tehran, Iran; 3 Human Genetics Research Center, Baqiyatallah University of Medical Sciences, Tehran, Iran

**Keywords:** Angiogenesis, Immunotherapy, Immunotoxin, Tumor, Vascular endothelial growth- factor

## Abstract

**Objective(s):**

A variety of signaling molecules have been identified that play a role in angiogenesis, of prime importance, vascular endothelial growth factor (VEGF) and its resceptor (VEGFR), which is highly expressed in most human solid tumors. Targeting VEGF or/and VEGFR with immunotoxin may be a promising approach to directly affect cancer cells. Immunotoxins are for targeted treatment comprising two functional moieties, an antibody that binds to target cells along with toxin that kills molecules.

**Materials and Methods:**

In this study, an immunotoxin comprising domain of diphtheria toxin subunit A (DT386) genetically fused to mouse VEGF (mVEGF-DT) was developed. The second construct, which contains the DT386 domain, was made to investigate the action of the DT386 domain on tumor cells. Both gene constructs were cloned, expressed, and were further purified. The biological activity of mVEGF-DT and DT386 proteins was assessed on the TC1 cell line bearing mouse model. Proteins were injected intra-tumoral in mice, in separate groups.

**Results:**

Tumors in the mVEGF-DT group started to dwindle after six injections, but tumor size in both control groups (DT386 and PBS), continued to grow.

**Conclusion:**

Successful targeting of solid tumor cells by mVEGF-DT immunotoxin demonstrates the therapeutic potential utility of these conjugates for tumor targeting.

## Introduction

Forty years ago, Folkman claimed that angiogenesis is essential for tumors to grow beyond essential size (1) and it is now proven to be an indisputable fact (2). Since then efforts have been made to suppress this physiological process, which is tightly regulated during human growth. Dysregulation of this process results in diabetic retinopathy and development of solid tumors (3, 4). It has been shown that vascular endothelial growth factor-A (VEGF-A) and vascular endothelial growth factor receptor2 (VEGFR2) are the predominant regulators of this process (5-9). 

Immunotoxins are a class of proteins that contain a toxin at one end and a cell-binding ligand at the other end. First immunotoxins were made by chemically conjugating an antibody to a toxin (10, 11). The next class of immunotoxins, antibody fragments were replaced by smaller molecules such as growth factors or cytokines (12). Three main toxin components of immunotoxins that are tested clinically are pseudomonas exotoxin (PE), diphtheria toxin (DT), and ricin (13). Examples of immunotoxins that have entered phase one clinical studies include anti-CD33, conjugated to gelonin (14), or anti-mesothelin variable antibody fragment [Fv] linked to PE (15). DT is synthesized in *Corynebacterium diphtheria* as a single-chain enzyme of 535 amino acids with a molecular weight of 63 kDa [1], [2]. This toxin has two domains namely A and B. B domain is responsible for cellular binding and translocation, while A subunit is responsible for inhibition of protein synthesis by transferring the ADP-ribosyl moiety of NAD^+^ to the eukaryotic polypeptide elongation factor 2 (EF2) (16, 17). DT protein which lacks the receptor-binding domain is non-toxic to human cells (18). This toxin has been used as an immunotoxin in several other fusion proteins such as DT386-BR2 (19), E7777 (20), and DT388-IL3 (21, 22). Among those, E7777 has entered phase one clinical trial (20). 

In this study, the diphtheria toxin subunit was genetically fused to mouse VEGF and formed an immunotoxin. This protein was expressed in the bacterial expression system and its biological activity was *in vivo* assessed in the mouse tumor model. 

## Materials and Methods

***Gene construction and cloning***

**Figure 1:**
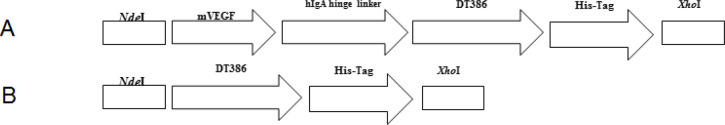
Schematic diagram of fusion proteins used in this study. A: MVEGF-DT immunotoxin. B: Diphtheria toxin (control protein)

Two DNA coding sequences consisting of mouse VEGF (NM_001025257.3) and truncated diphtheria toxin, DT386, genetically fused with a human IgA1 hinge as a linker and synthesized (Biomatik, Canada). DNA fragments were sub-cloned into the pET22b expression vector in *Nde*I and *Xho*I restriction sites of the vector following ligation by T4 DNA ligase and were transformed into *E. coli* TOP10F’. The resulting plasmid was named pET-mVEGF-DT. The cloning procedure was confirmed by colony-PCR with T7 universal primers and restriction enzyme digestion. The DT fragment was also cloned and constructed (entitled as pET-DT). Figure 1 shows the two constructs that were used in this study.

***Immunotoxin expression and purification***

BL-21 (DE3) bacteria were transformed with pET-mVEGF-DT and pET-DT plasmids. The single colony that was positive in colony PCR and digestion, was inoculated in LB media in presence of selective antibiotics. When OD_600_ of 0.6 was reached, expression of recombinant protein was induced with 1mM of Isopropyl-beta-d-thiogalactopyranoside (IPTG) and incubated at 37 °C overnight. Recombinant expression was confirmed by SDS-PAGE and Western blotting. Briefly, proteins were separated in 15% SDS-PAGE and were transferred to nitrocellulose paper with the semi-dry method (45 min, 15 volts). Blot was blocked by 3% skimmed milk overnight at 4 °C and then incubated with 1:2000 diluted rabbit anti-His antibody and anti-rabbit-HRP conjugated antibody (1:1000). Finally, the blot was developed with 4-chloro-1-naphthol.

Protein purification was performed under denaturation conditions. The cell pellet was collected by centrifugation (8000 rpm, 15 min) and re-suspended in the binding buffer (100 mM NaH_2_PO_4_, 10 mM Tris-Cl, 8 M Urea, pH 8). Sonication was used to lyse the cells. The soluble fraction was separated by centrifugation and 0.45-micrometer filter, and the lysate was applied to the Ni-NTA column (Qiagen, Germany) which was pre-equilibrated with binding buffer. After washing the column with wash buffer (100 mM NaH_2_PO_4_, 10 mM Tris-Cl, 8 M Urea, pH 6.3), His tagged portions were eluted by applying elution buffer (100 mM NaH_2_PO_4_, 10 mM Tris-Cl, 8 M Urea, pH 4.5). The purity of the preparation was verified by SDS-PAGE.

***In vivo*** **assay**

***Mouse and tumor model***

Three to four week old C57BL/6 (female) mice were purchased from the animal facility of Pasteur Institute of Iran. All animals used in this study were cared for according to the animal care and use protocol of the Pasteur Institute of Iran. The tumor was induced in these mice by injection of 10^6^ TC-1 cells (final volume of 200 µl in PBS). TC-1 was derived from primary lung epithelial cells of C57BL/6 mice. The right flank of the C57BL/6 mice was shaved, and cells were injected. This mouse was used as stock. Following the establishment of the tumor, the stock mouse was sacrificed and the tumor was dissected into 3 mm^3^ segments that were then transplanted into 18 mice. Tumor size was monitored three times a week using a caliper according to this equation (23): V=L×W2×0.52, where V: volume, L: length, and W: width. Tumor volume change was monitored according to the relative tumor volume (RTV) formula: tumor volume in day X / tumor volume in day 0.

***Treatment study***

The TC-1 tumor-bearing C57BL/6 mice were divided into three groups, each group contained 6 mice. Mice were injected intra-tumoral in weekly intervals. Treatment started when tumor volume reached 4 mm^3^ and continued for 2 months. The mortality was monitored during this period. For each injection, the control group received 100 µl PBS; the DT group received 100 µg of DT toxin, and the test group received 100 µg of mVEGF-DT. The final volume for each injection was 100 µl. 

***Ethical standards***

All animal studies have been approved by the Institutional Ethics Committee (Pasteur Institute of Iran). All researchers got acquainted with the ethical methods of working with laboratory animals before starting their research.

***Statistical analysis***

GraphPad PRISM software was used for statistical analysis. The survival rate was evaluated by Kaplan-Meier analysis. Two-way ANOVA was used for comparison. The mean of the size of the tumor was calculated within each group, and one-way ANOVA was used to determine the significant difference between groups (*P*<0.05). 

## Results

***Gene construction and expression***

**Figure 2:**
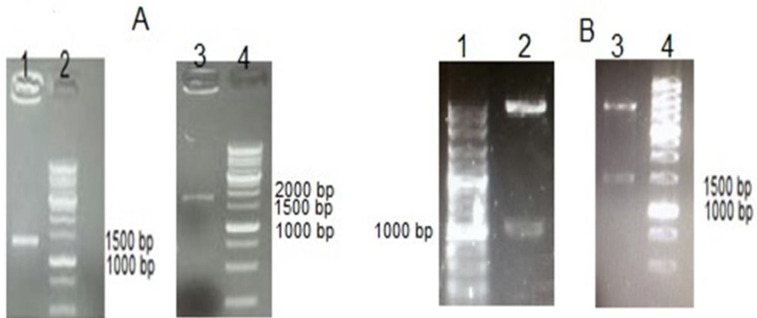
(A) Colony PCR of final construct with T7 primers. Lane 1; MVEGF-DT and Lane 3; DT, Lane 2, 4; 1kb DNA ladder. (B) Conformational digestion of mVEGF-DT and DT. Lane 2; pET-DT, Lane 3; pET-mVEGF-DT . Lane 2 , 4; 1kb DNA ladder. Cat No.PR901645

Recombinant pET-mVEGF-DT and pET-DT constructs were synthesized and sub-cloned to pET22b. The sub-cloning procedure was confirmed by colony-PCR with T7 primers (Figure 2A), *Nde*I, and *Xho*I digestion (Figure 2B).

**Figure 3:**
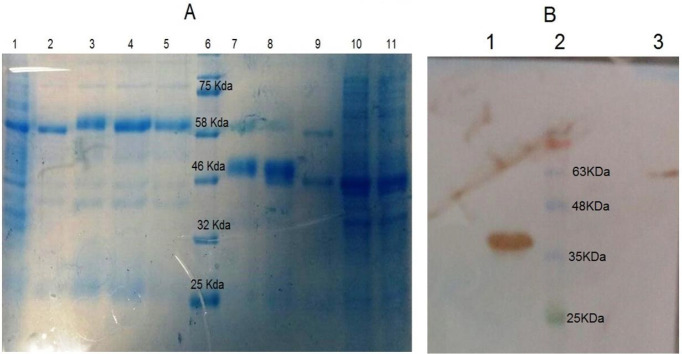
SDS-PAGE and Western blot. (A) SDS-PAGE (15%) of expressed and purified proteins. Lane 1; mVEGF-DT bacterial lysate, Lane 2-5; purified mVEGF-DT, Lane 6; protein marker Cat No PR901641, Lane 7-9; purified DT. Lane 10-11; DT bacterial lysate. (B) Anti-His Western blot analysis. Lane 1; DT protein, Lane 2; protein marker Cat No PR901641, Lane 3; mVEGF-DT protein

Expression of recombinant mVEGF-DT and DT was induced by 1mM of IPTG. Expression of mVEGF-DT and DT was confirmed by SDS-PAGE and Western-Blotting. Figure 3 shows the expression and purification of the proteins. A 43 KDa protein corresponding to DT protein and 58 KDa corresponding to mVEGF-DT was observed.

***In vivo treatment of tumor***

**Figure 4:**
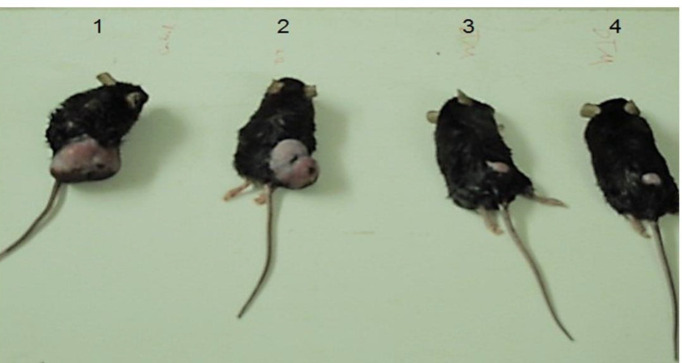
Therapeutic effect of mVEGF-DT on mice. On the 6th week of tumor implantation, mice were photographed. Difference in tumor size is noticeable. 1; PBS group, 2; DT group, 3; and 4; mVEGF-DT group

**Figure 5:**
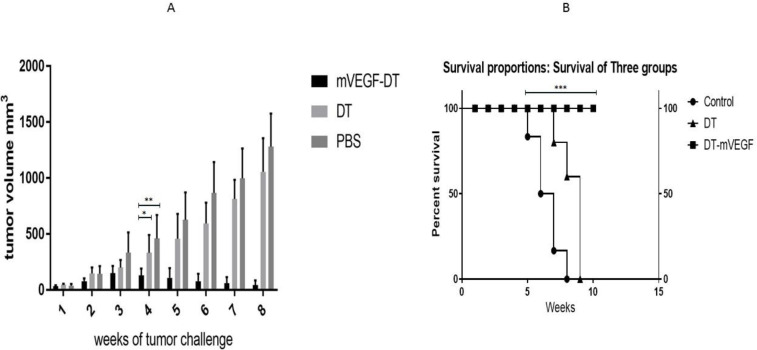
(A) Changes of tumor volume during treatment. Significant difference between MVEGF-DT with DT (*: *P*-value = 0.022) and PBS (**: *P*-value = 0.006) from the fourth week onwards was observed. (B)Kaplan-Meier survival curves of mice bearing TC1 induced tumor. Mice that were treated with MVEGF-DT had a prolonged survival rate (***: *P*-value <0.0001)

For *in vivo* treatment of tumor in mice, 18 C57BL/6 mice harboring TC1 cell-induced cancer were divided into three groups. The therapeutic effect of mVEGF-DT is shown in Figure 4. In the sixth week, tumor volumes were approximately the same size at the beginning of the experiment. All three groups showed a gradual increase in tumor volume within the first three weeks of treatment, whereas this increase in the PBS group was sharper than others. mVEGF-DT treated group displayed a substantial decrease in volume after 3 weeks of treatment, as compared with PBS and DT groups. Also, DT group showed a slower increase in tumor growth when compared with the PBS group. The difference between the three groups was significant from the second week (*P*-value = 0.003). There was a significant difference between the mVEGF-DT group and the DT group (*P*-value = 0.022) and also the mVEGF-DT group and the PBS group (*P*-value = 0.006) from the fourth week onwards. Also, there was no significant difference between the two groups of DT and PBS by the end of the eighth week (*P*-value = 0.499). All mice in the mVEGF-DT treated group survived until the end of the experiment (Figure 5). There was a significant difference between the three groups in the survival of mice (*P*-value<0.0001). DT protein had increased the survival of the tumor-bearing mice when compared with the PBS group (*P*-value<0.0003).

## Discussion

Immunotoxins include a target recognition moiety that is connected to bacterial or plant proteinaceous toxins. The target recognition portion can include complete monoclonal antibodies, antibody fragments, or ligands that bind to the receptor on the cell surface. Diphtheria toxin is one of the most widely used toxins for the development of immunotoxins (24, 25). It was first pointed by Yamaizumi that diphtheria toxin is toxic for mammalian cells (26), and it was Thorpe, who later discovered that conjugation of DT to the antibody can increase the efficiency of targeting (27). Diphtheria toxin consists of two subunits A and B. Fragment A is non-toxic outside the cell, but once it enters the cell by binding to elongation factor-2 (EF-2), it prevents protein synthesis and destroys the cell (28). Therefore, DTA can be a highly potent molecule for cancer therapy, if it can be delivered specifically into the tumor cells. To date, three immunotoxins have been FDA approved to be used in humans; interleukin -2 conjugated to the diphtheria toxins (Ontak^TM^) administered as an antineoplastic agent, interleukin -3 conjugated to the diphtheria toxin (Elzonris^TM^) used for the treatment of blastic plasmacytoid dendritic cell neoplasms (29) and anti-CD22 conjugated to Pseudomonas exotoxin A (Lumoxiti®) approved for the treatment of relapsed or refractory hairy cell leukemia (30). Immunotoxins are being successfully tested to treat many different diseases, like liver cancer (31), prostate cancer (31), pancreatic cancer (32), and autoimmune diseases (33). The specificity of the recombinant immunotoxins is determined by distribution location and expressing the extent of the targeted antigens. In some instances, the targets are presented on normal tissues as well as tumor cells, which could induce side effects by nonspecific binding. Compared with solid tumors, hematological tumors are more sensitive to immunotoxin therapy. 

Angiogenesis is a critical multi-step process that results in the growth and sprouting of solid tumors. It has been proven that inhibition of angiogenesis, is an effective way to treat solid tumors and is a key factor in the growth of solid tumors. This process is involved in many different physiological processes such as embryonic development and wound healing (34). Dysregulation of angiogenesis, on the other hand, leads to diabetic retinopathy and development and progress of solid tumors (3, 4). Therefore, inhibition of tumoral angiogenesis is one of the goals of cancer treatment. Angiogenesis is a complicated process by which new vessels are formed via stimulation of some small molecules, of prime importance, vascular endothelial growth factor (VEGF), and blockade of VEGF leads to inhibition of tumor angiogenesis (35). Some immunotoxins have been developed to target endothelial cells and therefore, inhibit tumor growths such as VEGF121-DT (36) and Shiga-like toxin-VEGF (37). An immunotoxin containing VEGF121/rGel was developed by Veenendaal *et al*., and it was shown that destruction of the vasculature around melanoma and human prostate carcinoma xenografts in mice leads to decreased tumor volume (38). They specifically showed that eradication of tumors was not because of the direct toxicity of VEGF121/rGel on tumors. In this study, we developed an immunotoxin that has the mouse VEGF on its N-terminal and subunit A of the diphtheria toxin at its C terminal, which is separated by a human IgA1 hinge as a linker. We showed our immunotoxin could successfully inhibit tumor growth in the mouse tumor model when compared with control groups. In 2010, Hu *et al*. (39) used a DNA construct that contained VEGF165-PE38 for cancer therapy. The construct was injected into the tumors of nude mice of the malignant glioma model and inhibition of the growth of tumor size, and inhibition of capillary-like structures in their CAM assays was shown. Previously, our group showed VEGFR2-specific Nanobody Pseudomonas exotoxin A conjugated could efficiently inhibit cell proliferation (40). DT386 that was used in this study, lacks the B subunit, which prevents the toxin from binding to the eukaryotic cell membrane. Using this form of toxin that only has the A subunit, has shown to be not cytotoxic (41). The same results were observed in our study, that is the DT group injected mice were alive and were not dead from DT386 side effects. The DT group showed an increase in the survival time in comparison with our negative (PBS) group. It can be because of the fact that our DT toxin was able to stimulate the response in mice and the hyperimmune mice escaped tumor mortality for a longer time.

## Conclusion

The successful targeting of solid tumor cells by mVEGF-DT immunotoxin demonstrates the therapeutic potential utility of these conjugates. Statistical analysis showed that a significant difference was observed between groups. However, it is suggested that the ability of the immunotoxin to detect VEGF be evaluated in flow cytometric and immunohistochemical tests. Furthermore, the level of immune response to immunotoxins in the animal model should be examined.

## Authors’ Contributions

MH, MB, BR, and RM Data analysis and draft manuscript preparation; NS and FK Critical revision of the paper; MB and MH Supervision of the research; HG and AO Data processing, collection, performing experiments; MB Study concept and design.

## Conflicts of Interest

The authors declare that they have no conflicts of interest.
